# The Extracellular Small Leucine-Rich Proteoglycan Biglycan Is a Key Player in Gastric Cancer Aggressiveness

**DOI:** 10.3390/cancers13061330

**Published:** 2021-03-16

**Authors:** Filipe Pinto, Liliana Santos-Ferreira, Marta T. Pinto, Catarina Gomes, Celso A. Reis

**Affiliations:** 1 Instituto de Investigação e Inovação em Saúde (i3S), University of Porto, 4200-135 Porto, Portugal; lilianaf@ipatimup.pt (L.S.-F.); mtpinto@i3s.up.pt (M.T.P.); cgomes@ipatimup.pt (C.G.); 2 Institute of Molecular Pathology and Immunology (IPATIMUP), University of Porto, 4200-135 Porto, Portugal; 3 Faculty of Sciences of the University of Porto (FCUP), University of Porto, 4169-007 Porto, Portugal; 4 Instituto de Ciências Biomédicas Abel Salazar (ICBAS), University of Porto, 4050-313 Porto, Portugal; 5 Faculty of Medicine of the University of Porto (FMUP), University of Porto, 4200-319 Porto, Portugal

**Keywords:** biglycan, small leucine-rich proteoglycan, gastric cancer, prognostic biomarker, cancer aggressiveness

## Abstract

**Simple Summary:**

Approximately 80% of gastric cancer patients are diagnosed at advanced stages with an average five-year survival rate of less than 30%. Alterations of the extracellular matrix proteins have been largely demonstrated in all steps of the disease. Thus, studies for the identification of novel prognostic biomarkers and efficient therapeutic strategies are urgently needed. In this study, we report the oncogenic role of biglycan, an extracellular proteoglycan, in gastric carcinogenesis. Biglycan was able to modulate gastric cancer aggressive features as cell survival, migration, and angiogenesis. Additionally, high levels of biglycan expression correlates with tumorigenic gene signatures and they are associated with poor patient prognosis in advanced stages of the disease. These results point biglycan as a key player in gastric cancer aggressiveness and further studies should be done to investigate the therapeutic potential of biglycan to tackle gastric cancer progression.

**Abstract:**

Biglycan (*BGN* gene), an extracellular proteoglycan, has been described to be associated with cancer aggressiveness. The purpose of this study was to clarify the clinical value of biglycan as a biomarker in multiple independent GC cohorts and determine the in vitro and in vivo role of biglycan in GC malignant features. We found that *BGN* is commonly over-expressed in all analyzed cohorts, being associated with disease relapse and poor prognosis in patients with advanced stages of disease. In vitro and in vivo experiments demonstrated that biglycan knock-out GC cells display major phenotypic changes with a lower cell survival, migration, and angiogenic potential when compared with biglycan expressing cells. Biglycan KO GC cells present increased levels of PARP1 and caspase-3 cleavage and a decreased expression of mesenchymal markers. Importantly, biglycan deficient GC cells that were supplemented with exogenous biglycan were able to restore biological features, such as survival, clonogenic and migratory capacities. Our in vitro and in vivo findings were validated in human GC samples, where *BGN* expression was associated with several oncogenic gene signatures that were associated with apoptosis, cell migration, invasion, and angiogenesis. This study provided new insights on biglycan role in GC that should be taken in consideration as a key cellular regulator with major impact in tumor progression and patients’ clinical outcome.

## 1. Introduction

Gastric cancer (GC) is the fifth most prevalent cancer and the third cause of cancer-related deaths worldwide [1]. Because of the lack of symptoms in the early-stage of the disease, GC patients are usually diagnosed at advance stages with presence of distant metastasis and subsequently associated with a dismal prognosis, with a five-year survival rate under 30% [2]. Several efforts have been made to classify gastric tumors in order to find novel biomarkers of prognosis as well as to guide treatment decisions. To date, the biomarkers most frequently used in the clinics is the serological assay CA19-9 [3,4,5], and the tumor tissue expression of HER2 or PD-L1 as biomarkers for therapy response [5,6,7]. However, their effectiveness in predicting patient outcome is still very poor [6]. To address this issue, it is crucial to identify key molecular players underlying GC tumor aggressiveness in order to better define patients’ prognosis and selection for appropriate therapeutic procedures.

The tumor microenvironment is one of the major common players that has been described to influence tumor initiation, development, and progression [8,9]. It is known that the extracellular matrix (ECM)-associated molecules, as the small leucine-rich proteoglycans (SLRP) [10,11], are deregulated in GC being associated with tumor aggressiveness, therapy resistance, and establishment of metastatic niches [12,13,14,15,16].

Biglycan (*BGN* gene) belongs to the class I of the SLRP family and it features a core protein with leucine-rich repeats with a molecular weight of 42 kDa [17]. However, when fully glycosylated, the molecular weight of biglycan increases up to 100–250 kDa. This is due to the presence of two chondroitin/dermatan sulfate (CS/DS) glycosaminoglycan (GAG) chains covalently attached to the protein [18]. This proteoglycan is ubiquitously expressed, with a pronounced expression in bone tissue, and it can be incorporated on the ECM or exist in the blood in its soluble form in disease conditions [18,19]. The biglycan clinical impact in cancer is still poorly understood and sometimes contradictory. For instance, in bladder cancer, it was demonstrated that high levels of biglycan predict poor prognosis of patients [20], while other studies correlated high levels of *BGN* mRNA with a favorable patient’s prognosis [21]. In colorectal cancer, high levels of biglycan have been associated with cancer aggressiveness, including tumor advanced stage, lymph node metastasis, and worse overall patient survival [22]. One of the major functions associated with biglycan expression in cancer is its potential to modulate cancer cell invasion, angiogenesis, and metastasis formation [23,24]. Biglycan was described to increase tissue stiffness, leading to an increase of melanoma invasiveness in vitro [25], and it was demonstrated that high levels of biglycan expression are able to promote angiogenesis as well as tumor cell intravasation and subsequent metastasis formation via TLR2/4 and ERK activation [26,27]. In GC, it has been shown that patients with high biglycan levels are associated with high tumor stages, vessel invasion, the presence of lymph node metastasis, and poor overall survival [28,29]. Hu et al. [29,30] showed that biglycan overexpression in GC cells increases in vitro invasion capacity when compared with biglycan negative controls. This aggressive phenotype was correlated with an increase of the focal adhesion FAK and Paxilin activation [29].

The aforementioned studies indicate an important role of biglycan in GC aggressiveness. However, they rely on the immature intracellular biglycan form (unglycosylated) underestimating the importance of the full glycosylated form and its role as an extracellular protein. The mature and functional active biglycan protein can be detected using the available antibodies after GAG removal by enzymatic digestion with chondroitinase ABC [31]. Indeed, the presence of a complex CS/DS GAG chains can hinder the antibody binding, leading to the misinterpretation of results. Real information regarding BGN expression and its functional role in GC biology is not fully understood due to the huge difficulty to study proteoglycans. In the present work, which combines both in silico, in vitro and in vivo approaches, we validated the clinical impact of *BGN* expression in GC patient samples, and we have established unique GC cellular models to study the effect of mature biglycan in GC aggressiveness.

## 2. Materials and Methods

### 2.1. Gastric Cancer Tissue Expression Analysis: Functional Annotation and Correlation Profiles

Gene expression data in GC patients were assessed in five independent GC cohorts (Chen (*n* = 112), Cho (*n* = 84), Cui (*n* = 160), Cho (*n* = 84), DErrico (*n* = 67), and Wang (*n* = 27)) from the Oncomine database [32], comprising a total of 450 tissues, with 174 adjacent normal tissues and 276 GC tissues. A comparison analysis of the most differential expressed genes in GC *versus* normal samples was performed in more than 10,700 genes. The mRNA *BGN* transcript levels were also investigated in the stomach cancer cohort (STAD) from The Cancer Genome Atlas (TCGA), comprising 408 cancer patients and 211 normal gastric tissues available at the Gene Expression Profiling Interactive Analysis (GEPIA) database [33].

The expression profiles of genes that were associated with *BGN* in GC samples (*n* = 408) were extracted from TCGA (STAD-TCGA) using the cBioPortal for Cancer Genomics [34,35] Genes with Spearman > 0.3 and *p* value < 0.001 were considered to be positively correlated with *BGN* (*n* = 2376 genes). Genes with Spearman < 0.3 and *p* value < 0.001 were considered to be inversely correlated with *BGN* (*n* = 817 genes). *BGN* positively correlated or inversely correlated gene profiles were subjected to Kyoto Encyclopedia of Genes and Genomes (KEGG) pathway analysis and Gene Ontology functional annotation analysis using the DAVID 6.8 tool [36], as described [37]. The pathways and biological clusters were considered to be significant when there was > 2-fold enrichment and *p* < 0.05. Correlation between *BGN*, epithelial (*CDH1, CKR19*), mesenchymal (*VIM, SNAI1, SNAI2, TGFB1, MMP2, MMP14, CDH2, ZEB1, ZEB2, FN*), anti-apoptotic (*BCL2, BCL2L2, BCL2A1, IQSEC2, BCL2L1)*, pro-apoptotic (CASP3, *CASP6, CASP5, CASP8, CASP10, FASN, BAK, BIK*), angiogenic (*VEGFB, VEGFC, KDR, FLT1, FGF2, PDGFC, ANGPT1, ANGPT2, ANGPTL2, ANGPTL1, ANGPTL4, ANGPTL7*), and extracellular matrix (*VCAM1, SELE, ITGA11, ITGA5, ITGB5, ITGBL1, LAMA2, LAMA4, LAMB2, COL1A1, COL1A2, COL4A2*) markers was performed using the GEPIA database [33]. RNA sequencing data (represented as log2 transcripts per kilobase million) of 408 GC samples (STAD, TCGA) were analyzed.

### 2.2. Clinical and Prognostic Impact of Biglycan in Gastric Cancer

We have used the Kaplan–Meier Plotter (KM plotter) database [38] with expression data for 1065 GC patients and survival data for 882 GC patients (GSE14210, GSE15459, GSE22377, GSE29272, GSE51105, and GSE62254) to determine the clinical relevance of *BGN* expression in GC. The patients were stratified in two groups with high and low expression. The top 33.3% (third tercile) of patient samples with the highest expression were considered as the high *BGN* expression group. The bottom 33.3% of patients with the lowest expression values (first tercile) were considered as the low *BGN* expression group. The overall survival was analyzed for all gastric cancer patients and then divided by stage (II *n* = 145; III *n* = 319; and, IV *n* = 152) and Lauren subtype classification (intestinal *n* = 336, diffuse *n* = 248, and mixed *n* = 33). The time to first progression (*n*= 645) and post-progression (*n*= 505) survival was also evaluated. The prognostic value of *BGN* expression was further validated in the Chen cohort (*n*= 84) and TCGA dataset (STAD, *n*= 378).

The available clinic-pathological data, including Lauren classification and TCGA molecular subtype, stage, presence of lymph node invasion, metastasis, and recurrence, were also extracted from five GC cohorts (DErrico, Chen, Cho, Takeno [32], and STAD-TCGA) and compared with the *BGN* expression levels.

### 2.3. Cell Culture

Four GC cell lines (NCI-N87, AGS, MKN45, and MKN74), one colorectal cancer cell line (Caco-2), and one human embryonic kidney cell line (HEK-293) were used in this study. NCI-N87, AGS, MKN74, and Caco-2 cell lines were obtained from the American Type Culture Collection (ATCC; Manassas, VA, USA) and MKN45 and HEK-293 cell lines were obtained from the Japanese Cancer Research Bank (Tsukuba, Japan). All of the cells were grown in monolayer and maintained at 37 °C in a humidified chamber with 5% CO2. NCI-N87, AGS, MKN45, and MKN74 cell lines were maintained in Roswell Park Memorial Institute 1640 GlutaMAX medium (RPMI; Gibco, Thermo Fisher Scientific, Waltham, MA, USA), whereas Caco-2 and HEK-293 cells were maintained in Dulbecco’s Modified Eagle Medium (DMEM, high glycose/pyruvate; Gibco). The media were supplemented with 10% fetal bovine serum (FBS, Biowest, Riverside, MO, USA) and replaced every 2–3 days. The authentication of cell lines was performed by short tandem repeat (STR) DNA typing according to the International Reference Standard for Authentication of Human Cell Lines. All cell lines were checked for the absence of mycoplasm infection. 

### 2.4. Quantitative Reverse Transcriptase PCR (qPCR)

Total RNA was extracted from cell lines using TRI Reagent (Trizol, Sigma-Aldrich, St. Louis, MO, USA). One μg of RNA was reverse transcribed with random primers using the SuperScript^®^ IV Reverse Transcriptase Kit (Invitrogen, Thermo Fisher Scientific, Waltham, MA, USA). RT-qPCR was performed with 1 μL of cDNA, 10 μM of each primer, 10 μL SYBR^®^ Green Master Mix (1×) (Thermo Fischer Scientific) and ultrapure water to a final volume of 20 μL using the ABI 7500 (Applied Biosystems, Thermo Fisher Scientific, Waltham, MA, USA). The following primers were used, *BGN* (Fw: 5’-AAGGTGCCCAAGGGAGTGTTC-3’; Rv: 5’-TGGTCTAGGTGGAGTTCATTCAGG-3’), *CDH1* (Fw: 5’-ATTTTTCCCTCGACACCCGAT-3’; Rv: 5’-TCCCAGGCGTAGACCAAGA-3’), *CDH2* (Fw: CCATCAAGCCTGTGGGAATC-3’; Rv: GCAGATCGGACCGGATACTG-3’), *ZEB1* (Fw: 5’-CAGCTTGATACCTGTGAATGGG-3’; Rv: 5’-TATCTGTGGTCGTGTGGGACT-3’), *ZEB2* (Fw: 5’-GGAGACGAGTCCAGCTAGTGT-3’; Rv: 5’-CCACTCCACCCTCCCTTATTTC-3’), *SNAI1* (Fw: 5’-ACTGCAACAAGGAATACCTCAG-3’; Rv: 5’-GCACTGGTACTTCTTGACATCTG-3’), *NANOG* (Fw: 5’-TCTCCTCTTCCTTCCTCCAT-3’; Rv: 5’-CCTTGTCTTCCTTTTTTGCG-3’), *POU5F1* (Fw: 5’-AGCAAAACCCGGAGGAGT; Rv: 5’-CCACATCGGCCTGTGTATATC-3’), *DPP4* (Fw: 5’-AGTGGCACGGCAACACATT; Rv: 5’-AGAGCTTCTATCCCGATGACTT-3’). Relative gene expression was normalized to β-actin (Fw: 5’-AGAAAATCTGGCACCACACC-3’; Rv: 5’-TAGCACAGCCTGGATAGCAA-3’) by ΔCT (relative expression). Three independent experiments with three technical replicates per condition were performed.

### 2.5. Biglycan CRISPR/Cas9 Knock-Out

Biglycan knock-out was performed using CRISPR/Cas 9 technology, as described previously [39,40]. Briefly, six different gRNAs (gRNA1: TATCTGTCCGGTGTGTCCGG; gRNA2: ACACCGGACAGATAGACGTG; gRNA3: GCCATTCATGATGAACGATG; gRNA4: GGCCTGTGTGGCCTGTCTAT; gRNA5: CCGGACACACCGGACAGATA; gRNA6: TTTCGGTCCGCCGGACACAC) were designed using deskgen platform (https://www.deskgen.com/landing/ accessed on 12 February 2020) and then validated in HEK-293 cell lines. The gRNA5 was selected for plasmid production (Figure S1). After cell transfection, single cell sorting was performed to obtain different clones. Two biglycan knock-out clones presenting different indel mutations (BGN KO.5, and KO.12) were selected for the following experiments.

### 2.6. Total Protein Extraction and Secretome Protein Enrichment

The total protein cell lysates were obtained from GC cell lines and Caco-2 cell line. Briefly, the cells were plated into a six-well plate and when they reached 70% confluence, the cells were washed with cold PBS 1X and then scrapped in lysis buffer 17 (R&D Systems, Minneapolis, MN, USA) supplemented with complete TM protease inhibitor cocktail (Roche, Basel, Switzerland) and with PhosSTOP phosphatase inhibitor cocktail (Roche) for a 1.5 mL eppendorf tube. After incubation on ice for 30 min., cell supernatant was collected for a new eppendorf after centrifugion at 10,000 rpm for 10 min. The total protein concentration was determined using the DC protein assay (BioRad, Hercules, CA, USA) that was measured at 750 nm with Synergy™ Mx Monochromator-Based Multi-Mode Microplate Reader (BioTek Instruments, Inc., Winooski, VA, USA). The total protein extracts were also extracted from cells treated for 4 h with exogenous biglycan (5 μg/mL commercial purified BGN protein; B8041; Sigma-Aldrich). For secretome protein enrichment, each cell line was seeded in a T25 flask in normal medium (supplemented with 10% FBS). In the next day, cells were washed five times in PBS 1X and leaved in serum-free culture medium for 72 h. The conditioned media (secretome) was centrifuged for 5 min. at 1200 rpm to remove cell debris and concentrated using 30 kDa Amicon Ultra-4 centrifugal filter units (Merck Millipore, Burlington, MA, USA). The concentrated secretome was then washed with PBS 1X and subjected to another centrifugal step for 15 min. at 4000 rpm at room temperature. Lastly 4 μL of completeTM protease inhibitor cocktail (Roche) was added per 100 μL of concentrated samples and then stored at −80 °C. 

### 2.7. Enzymatic Chondroitinase ABC Deglycosylation Digestion

Chondroitin sulfate chains (CS GAGs) were removed from total cell lysates (20 μg) and secretome (15 μL) samples by chondroitinase ABC enzymatic treatment (chABC). The samples were digested with 10 mU of chondroitinase ABC protease free from *Proteus vulgaris* (chABC; AMS.E1028-02, AMS Biotechnology Limited, Oxfordshire, UK). The digestion reaction performed in adequate buffer, Tris-HCl, pH 7.5 (20 mM), and sodium acetate (20 mM) [41], in a final volume of 20 μL overnight at 37 °C. The deglycosylated protein samples were loaded onto 10% SDS–PAGE and then immunoblotting was performed. 

### 2.8. Western Blot

The protein samples (20 μg) were denatured and reduced for 5 min. at 95 °C in Laemmli Sample Buffer (1X; Bio-Rad) containing 10% of the reducing agent β-mercaptoethanol (Sigma-Aldrich). The samples were resolved in standard 10 % SDS-PAGE gels or in 4–15 % polyacrylamide gradient precast gels (Mini-PROTEAN^®^TGX™; BioRad) for cell death-related protein analysis. Membranes were probed with primary antibodies overnight at 4 °C. The list of antibodies contained the following: anti-biglycan (1:1000, HPA003157, Sigma-Aldrich), anti-chondroitine 4 sulfate (C4S–1:1000, BE-123, Sigma-Aldrich), anti-cleaved caspase 3 (1:1000, 9661,Cell Signaling Technology, Leiden, The Netherlands), anti-PARP1/cleaved-PARP1(1:1000, sc-7150, Santa Cruz Biotechnology, Heidelberg, Germany), anti-E-cadherin (1:1000, 4A2C7, Thermo Fisher), anti-N-cadherin (1:300, sc-59987, Santa Cruz Biotechnology), anti-Nanog (1:1000, ab77095, Abcam), anti-β -actin (1:1000, 13E5, Cell Signaling Technology), and anti-α-tubulin (1:10,000, DM1A, Sigma-Aldrich). Peroxidase-conjugated secondary antibodies were used according with the primary antibody isotypes and host species: goat anti-mouse IgG at 1:5000 (H+L), goat anti-rabbit IgG at 1: 15,000 (H+L), or goat anti-mouse IgG1 at 1: 25000 (all from Jackson ImmunoResearch, West Grove, PA, USA), and blot detection was done by chemiluminescence using ECL™ Western Blotting Detection Reagent and Amersham™ Hyperfilm™ ECL (both from GE Healthcare Life Sciences, Chicago, IL, USA). Protein expression quantification was performed while using ImageJ software (version 1.53c).

### 2.9. Immunofluorescence

The cells were grown in 12 well chamber glass slides (IBIDI, Martinsried, Germany) until 60 % of confluence, washed with PBS 1X, and then fixed in 4% paraformaldehyde (Alfa Aesar, Kandel, Germany) for 10 min. Unspecific secondary antibody detection was blocked using goat serum (DAKO, Agilent, Santa Clara, CA, USA) diluted 1:5 in 10 % BSA in PBS for 40 min at room temperature, followed by primary antibody incubation overnight at 4 °C. The primary antibodies were used in the following dilutions: anti-cleaved caspase 3 clone 9661 at 1:200 (Cell Signaling Technology), anti-E-cadherin clone 4A2C7 at 1:100 (Thermo Fisher), and anti-Ki67 clone MIB-1 at 1:50 (DAKO). After incubation with primary antibody, the slides were washed and incubated for 45 min. with respective goat secondary antibodies: anti-mouse Alexa Fluor^®^ 488-conjugated or anti-rabbit Alexa Fluor^®^ 488-conjugated (Thermo Fisher Scientific). For cytoskeletal (actin filaments) staining, the slides were incubated with Alexa Fluor^®^ 568 phalloidin (Invitrogen, Thermo Fisher Scientific) and the nuclei were counter stained with DAPI (4′,6-diamidino-2-phenylindole; Sigma-Aldrich). Finally, the slides were mounted using VectaShield mounting medium for fluorescence (Vector Laboratories, Burlingame, CA, USA) and then visualized by Zeiss Imager Z.1 microscope (Zeiss, Oberkochen, Germany). The images were acquired using a Zeiss Axio cam MRm and the AxioVision Rel. 4.8 software (both from Zeiss).

### 2.10. Trypan Blue Exclusion Assay

The trypan blue cell staining allows for the discrimination between viable and non-viable cells. Cells (1.5 × 10^5^ cells/mL) were plated in 12-well plates and then allowed to grow for 24 h, 48 h, or 72 h. At the end of each time point, cells and respective medium were collected and centrifuged at 1200 rpm for 5 min. 10 uL of each cell suspension was diluted in 0.4 % trypan blue dye (Gibco, Thermo Fisher Scientific) in a 1:1 ratio and the viable (unstained) and nonviable/dead (stained) cells were counted in a hemocytometer chamber.

### 2.11. Colony Formation Assay

Colony formation assay was used to assess the survival capacity of gastric cancer cells with and without BGN expression/stimulation. Cells (0.1 × 10^3^ cells/well) were plated in a six-well plate in: (i) 10% FBS RPMI medium; (ii) 0.1% FBS RPMI medium; (iii) 5 μg/mL commercial purified BGN protein (B8041; Sigma-Aldrich) in 0.1% FBS RPMI medium; or, with iv) BGN expressing cells secretome (2X concentrated). Medium and treatments were replaced every 3–4 days. After 15 days of culture, the plates were washed twice with PBS1 X and formed colonies fixed and stained in a mixture of 1% methanol with 0.5% crystal violet solution for 15 min [38,39]. The colonies formed were manually counted. 

### 2.12. Wound-Healing Migration Assay

The cells were seeded in 12-well plates and cultured to at least 95% of confluence. The monolayer cells were washed with PBS 1X, scraped with a plastic 200 μL pipette tip, and then incubated with: (i) 10% FBS RPMI medium; (ii) 0.1% FBS RPMI medium; (iii) 5 μg/mL commercial purified BGN protein (in 0.1% FBS RPMI); or. with (iv) BGN expressing cells secretome (2X concentrated). The ‘‘wounded’’ areas were photographed by phase contrast microscopy at different time points. The relative migration distance was calculated, as already described [37,42].

### 2.13. In Vivo Angiogenic Chicken Embryo Choriallantoic Membrane (CAM) Assay

The CAM model was used to evaluate the angiogenic response and the tumor size formation of the xenografted tumor cells from of GC WT and BGN KO cells, as previously described [43,44,45,46]. 

Fertilized chick (*Gallus gallus*) eggs that were obtained from commercial sources (Pintobar, Braga, Portugal) were incubated at 38 °C and 70% humidity. On embryonic development day 10, 1 × 10^6^ cells of each cell model were inoculated in a 1:1 suspension with matrigel per embryo on a 5 mm diameter nylon ring that was placed on top of the growing chicken CAM under sterile conditions. After four days’ post-inoculation, the CAMs were removed from the embryos, fixed in vivo with 4% paraformaldehyde, and placed on transparent plastic plate. Ex ovo digital images of CAM were acquired under a stereoscope at 20× magnification (Olympus, SZX16 coupled with a DP71 camera). The number of new vessels growing towards the inoculation site, delimited by the ring mark, were counted (less than 20 μm in diameter) and the area of the tumors (dense areas) were determined using the Cell^A Olympus program, as described [44,45].

### 2.14. Immunohsitochemical Analysis

Paraffin-embedded sections of excised formalin-fixed CAMs were stained with hematoxylin and eosin (H&E) for histological examination. Histological slides with 4 µm-thick tissue sections were subjected to immunohistochemistry analysis according to the modified avidin-biotin-peroxidase complex (ABC) method (Vector Laboratories, Burlingame, CA, USA) while using the monoclonal antibodies anti-Ki-67 (1:100; MIB-1, DAKO) or anti-cytokeratin AE1/AE3 (1:300; #50-9003-82; Invitrogen, Thermo Fisher Scientific), as described [46]; or, the polyclonal antibody anti-biglycan (1:100, HPA003157, Sigma-Aldrich). Four GC tissues were used to visualize biglycan sublocalization. Digital pictures were taken under 200X magnification with Zeiss Optical Microscope (Zeiss).

### 2.15. Statistical Analysis

Cumulative survival probabilities were calculated using the Kaplan–Meier method. Differences between the survival rates were evaluated by univariate log-rank test and the hazard ratio (HR) with 95% confidence interval was calculated. Multivariate survival analysis (Cox proportional hazard model, adjusted for the patient age, gender, and tumor stage) was performed in the STAD-TCGA cohort. Linear regression model (Pearson R) was used to evaluate the correlation between gene expression profiles. For simple comparisons, two different conditions were analyzed using Student’s t test and 2-way ANOVA analysis (Benforroni post-test) for a comparison of two conditions over time using Prism GraphPad Prism v7.0. The level of significance in the statistical analyses is indicated as * = *p* < 0.05, as ** = *p* < 0.01, or as *** = *p* < 0.001.

## 3. Results

### 3.1. BGN Is Commonly Over-Expressed in GC and Predict Poor Prognosis in Advanced GC Patients

**Figure 1:**
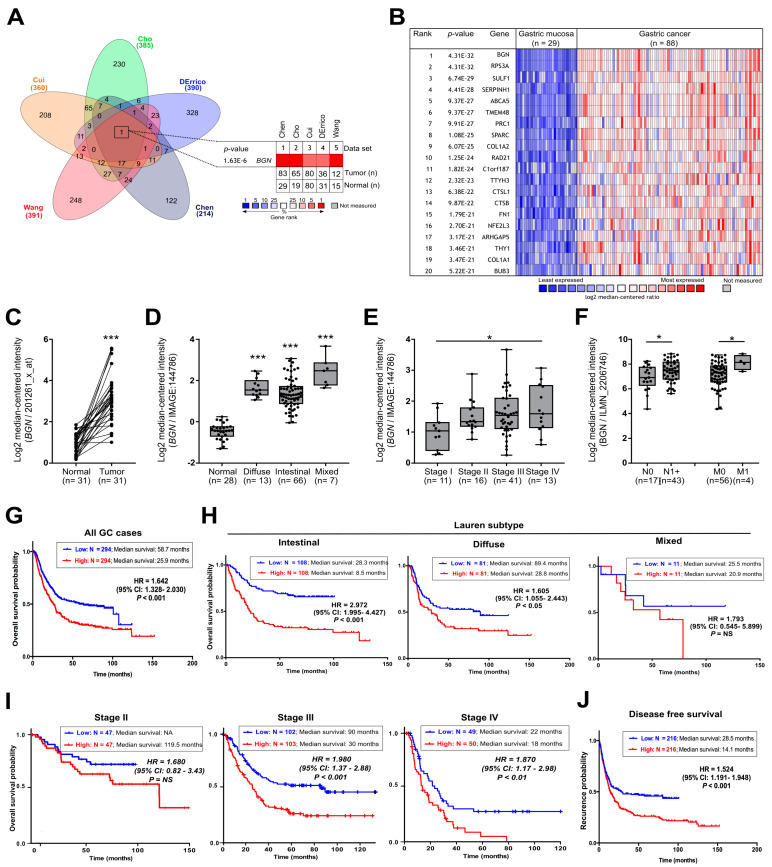
*BGN* (*biglycan*) is commonly over-expressed in gastric cancer (GC) and it is associated with tumor aggressiveness. (**A**) Venn diagram of the top 2% of genes most differential expressed in five independent GC cohorts from Oncomine database. (**B**) Heatmap of the top 20 genes more differential expressed in GC *versus* normal gastric mucosa from Chen cohort. *BGN* is the top 1 gene in the list. (**C**) *BGN* expression in patient tumor and normal paired-wise samples (DErrico cohort). (**D**) *BGN* expression according to Lauren classification (Chen cohort). (**E**) *BGN* expression by tumor stage (Chen cohort). (**F**) *BGN* mRNA levels are elevated in patients with presence of lymph node invasion (N1+) and metastasis (M1) (Cho cohort). (**G**) Kaplan–Meier analysis between *BGN* mRNA levels (GEO cohort from KMPlotter database) and GC overall survival. The categorization of patients’ samples was assigned into low (first tercile, lowest 33.3%) and high (third tercile, highest 33.3%) subgroups according to the levels of *BGN* mRNA expression. (**H**) Survival analysis according to Lauren classification. (**I**) Survival analysis by GC stage classification. (**J**) Recurrence probability/time of progression free-survival. Hazard ratios (HR) with 95% confidence intervals (CI) are shown. *, *p* value < 0.05; ***, *p* value < 0.001.

Biglycan has been reported to be up-regulated in GC patients with potential as a new prognostic biomarker [28,29]. However, to be included into the clinical practice, it is strong evidence is necessary that supports its expression as a common event in GC progression and that its detection will improve patient management and outcome. As such, we start by exploring the transcriptomic profile of GC patients (*n* = 226) vs. normal gastric samples (*n* = 174) from five independent Oncomine GC cohorts. More than 10 000 genes were included in the analysis. For each cohort, we identify the GC up-regulated genes in comparison to normal adjacent tissues. For each cohort, the top 2% highly expressed genes were selected and compared between cohorts (Figure 1A). We found that *BGN* is the only gene that is common to all of the analyzed cohorts (Figure 1A), and it is the top 1 gene highly expressed in GC (Figure 1B). These results were further validated in the STAD-TCGA cohort (Figure S2A). At the protein level, we found that biglycan is mostly absent in normal gastric glands being overexpressed in metaplasia and gastric tumor tissues (Figure S2B). Biglycan was often observed in the cancer cell cytoplasm and in the stroma of gastric tumors (Figure S2B). Importantly, when comparing paired-wise tumor and normal samples from the same patients, we observed an up-regulation of *BGN* expression levels in all studied patients (Figure 1C and Figure S2C). Clinically, high levels of *BGN* were independent of the Lauren classification (Figure 1D and Figure S2D) and the TCGA molecular subtypes (Figure S2E), but it was associated with the more aggressive tumor stages (Figure 1E and Figure S2F,G). Elevated levels of *BGN* start to be noticed at stage IB (Figure S2H), classified by the presence of cancer cells in the proximal lymph nodes (in the outer muscular layers of the stomach), which may indicate an early event in the invasion and metastatic process. Indeed, we further found that high mRNA levels of *BGN* are associated with the presence of cancer cells in the lymph nodes, presence of metastasis (Figure 1F and Figure S2I), and tumor recurrence (Figure S2J).

We performed survival analysis in three distinct GC cohorts to further investigate and validate the impact of *BGN* expression in GC patient’s prognosis (Gene Expression Omnibus -GEO, *n*= 882; STAD-TCGA, *n*= 378; Chen dataset, *n*= 84). Independent of the analyzed cohort, patients with high levels of *BGN* expression had significantly shorter overall survival than patients whose tumors expressed low *BGN* levels (Figure 1G and Figure S2K). We divided the patients based on Lauren classification and tumor stage to verify that the prognostic value of *BGN* is not been influenced by the most relevant clinic-pathological features. We found that the *BGN* prognostic value is independent of Lauren classification (Figure 1H). However, we found that high levels of *BGN* predict poor prognosis in GC patient diagnosed with advanced stages of the disease (stage III: HR=1.98, 95% CI = 1.37–2.88, *p* value < 0.001; stage IV: HR = 1.87, 95% CI = 1.17–2.98, *p* value = 0.008) (Figure 1I), also being associated with tumor recurrence (HR = 1.52, 95% CI = 1.19–1.95, *p* value < 0.001) (Figure 1J) and post-progression survival (HR = 2.16, 95% CI = 1.66–2.81, *p* value < 0.001) (Figure S2L). It is important to note that, in the multivariate analysis, the association between *BGN* and overall survival was independent of other clinicopathological variables (stage, age, gender) (Table 1).

### 3.2. BGN Gene Profiling in GC Patients: Functional Annotation

**Figure 2:**
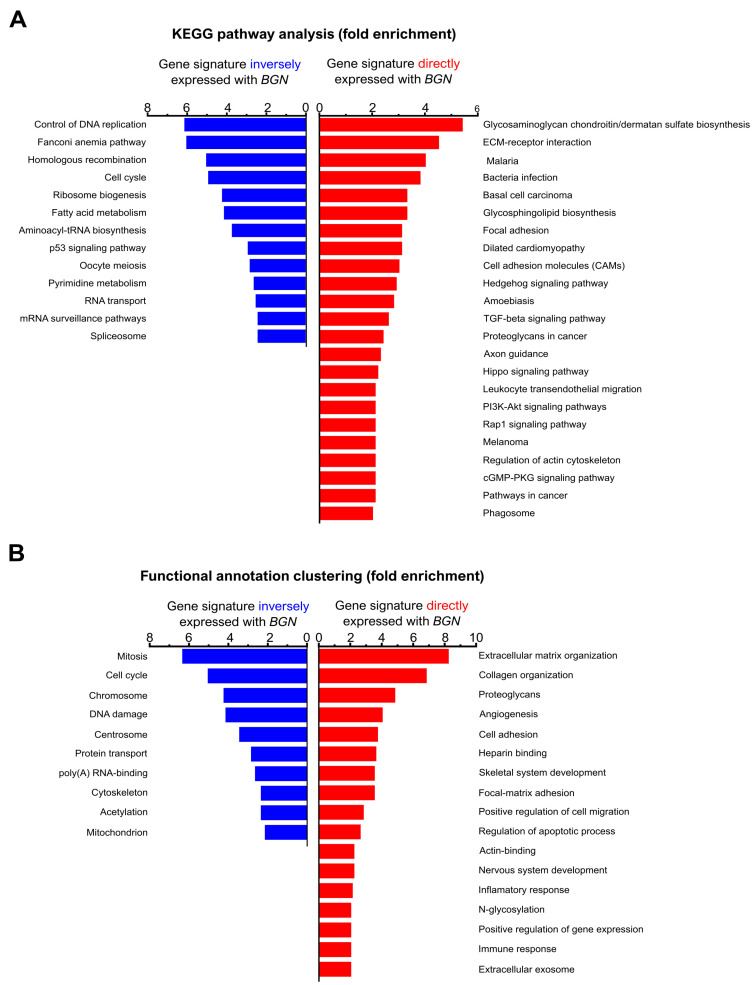
Functional analysis of *BGN* (*biglycan*) gene expression in clinical GC samples. (**A**) KEGG pathway analysis of genes profiles inversely expressed with *BGN* (blue) or directly coexpressed with *BGN* (red). (**B**) Classification of BGN gene-associated profiles into functional processes using the DAVID classification system. A minimum two-fold threshold and a *p* value < 0.05 was considered.

Our data indicate that *BGN* plays a crucial role in GC malignancy. As such, we explored the gene profile that is associated with the presence of *BGN* (coexpression) and the gene profile inversely associated with *BGN* expression in GC patients. Gene expression analysis was performed in 308 stomach cancer patients from TCGA (STAD-TCGA) while using the cBioPortal database. The *BGN* coexpression gene signature (Spearman R > 0.3 and *p* value < 0.001) and the gene signature inversely expressed with *BGN* (Spearman R< −0.3 and *p* value < 0.001) were extracted and then clustered based on the KEGG and functional annotation pathways using the DAVID 6.8 tool (Figure 2).

We found that the genes that are associated with low levels of *BGN* are involved in cell metabolism and cell cycle regulation processes (Figure 2A,B, blue). On the other hand, the gene signature coexpressed with *BGN*, which reflects the majority of GC patients, shown to be enriched in several oncogenic pathways, such as extracellular matrix regulation, angiogenesis, apoptosis, cell adhesion, and positive regulation of cell migration, proteoglycans, focal matrix adhesion, and heparin binding (Figure 2A,B, red). Knowing that biglycan is a proteoglycan and having in mind the important role of proteoglycans, for instance, in matrix–cell interaction and the regulation of signaling pathways, we may envision that *BGN* expression in GC is important to cell migration, cell death, angiogenesis, and the regulation of the extracellular matrix binding/organization. Thus, we want to further explore this hypothesis.

### 3.3. GC Cell Line Characterization of Secreted Biglycan and Establishment of Biglycan Knock Out Cell Models

Four GC cell lines (MKN74, MKN45, NCI-N87 and AGS) were characterized for biglycan expression to understand the biological role of biglycan in GC.

**Figure 3:**
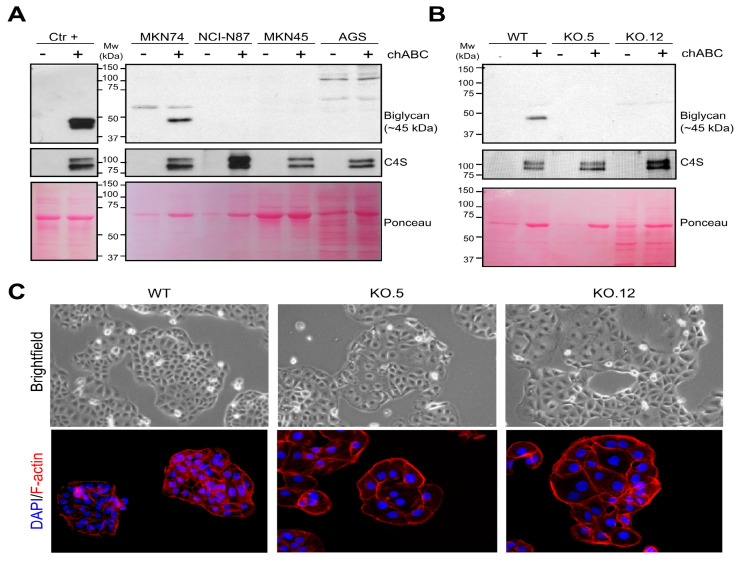
Characterization of biglycan expression in different GC cell lines and in knock out (KO) cells. (**A**) Biglycan expression in secretome samples from an intestinal cancer cell line Caco-2 (positive control) and from four GC cell lines (MKN74, MKN45, NCI.N87, and AGS) with and without chondroitinase ABC (chABC) treatment. Western blot analysis show MKN74 as the only GC cell line positive for biglycan. (**B**) Biglycan immunodetection in the MKN74 WT and KO cells (clones KO.5 and KO.12), showing the loss of expression in both KO clones. Chondroitinase-4-sulfate (C4S) immunedetection was used as a positive control for the chABC enzymatic treatment in all analysis. An increase in the C4S detection is expected after glycosaminoglycan (GAG) digestion by chABC. Ponceau staining was used as a loading control for secretome samples. (**C**) Bright field microscopy pictures (Magnification at 400×) and F-actin (red—phaloidin) immunofluorescence in MKN74 WT and biglycan KO cell (clones KO.5 and KO.12). Biglycan KO cells present morphological differences when compared with WT cells.

Biglycan core protein is described to have approximately 42 kDa [17]. However, biglycan, when fully glycosylated (functional protein), has two chondroitine sulfate GAGs that are attached to its core protein, increasing its molecular weight up to 100–200 kDa [18]. It has been difficult to assess biglycan expression at the protein level due to GAG complex structures and high molecular weight. To overcome this limitation, we performed a chondroitinase ABC (chABC) enzymatic treatment that is able to cleave and remove GAG chains. Using a positive control cell line Caco-2 (intestinal carcinoma cell line), it was possible to observe an enrichment in the detection of biglycan (42 kDa) in total cell lysates after chABC treatment (Figures S3A and S4). This result indicates that biglycan immunodetection can be significantly increased by GAG removal. However, we were not able to detect biglycan in total cell lysates from any of the GC cell lines studied (Figure S3A). Because the functional biglycan protein (fully glycosylated) is secreted to the extracellular matrix thus existing in its soluble form, we decided to evaluate the secretome of the GC cell lines while using Caco-2 as positive control. We observed that, after GAG removal, it was possible to detect a band at 42 kDa in the Caco-2 control cell line and in MKN74 cells, with no detection in any of the other GC cell lines studied (Figure 3A and Figure S4). This result is in agreement with the mRNA *BGN* levels assessed by real-time PCR (Figure S3B). The imunodetection of chondroitine 4-sulfated (C-4-S) unsaturated disaccharide neoepitopes, produced after the chABC digestion, was used as treatment control (Figure 3A and Figure S4). These results show that MKN74 GC cell line expresses a secreted fully glycosylated biglycan form thus being selected as a main cellular model to study the role of biglycan in GC malignancy.

Biglycan KO models were established using the CRISPR/cas9 system (Figure S1). We were able to obtain two biglycan KO clones (KO.5 and KO.12) that were identified at the genomic level by IDAA (Indel detection by amplicon analysis) [39,40] profiling analysis (Figure S3C) and validated by Sanger DNA sequencing (Figure S3D). Biglycan KO.5 has a two base pairs deletion and BGN KO.12 has an insertion of 152 base pairs when compared with WT profile. The presence of these indels results in a total absence of secreted biglycan expression in both KO5 and KO.12 models (Figure 3B). Phenotypically, the KO models showed dramatic changes in the cell morphology when compared with WT cells (Figure 3C). By brightfield microscope, biglycan KO cells seem to be larger when comparing with the WT cells. This result was validated by F-actin immunostaining, clearly showing that BGN KO cells are bigger as compared with WT cells, while maintaining cell–cell adhesion contact. This result is very interesting; nevertheless, we cannot exclude a morphological alteration as a clonal effect.

For further studies, the negative GC cell lines (NCI-N87 and MKN45) were also used to assess the role of exogenous biglycan in cancer aggressive features.

### 3.4. Biglycan Modulates GC Cell Migration through the Regulation of Epithelial-to-Mesenchymal (EMT) Markers

**Figure 4:**
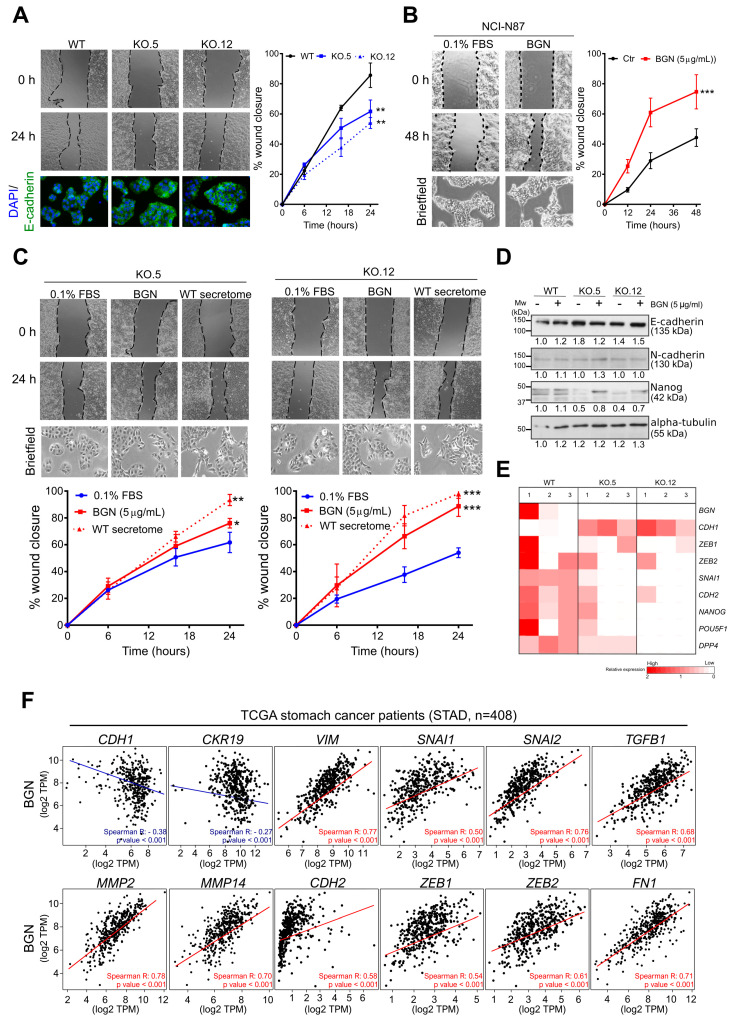
Biglycan modulates GC cell migration through the regulation of epithelial-mesenchymal (EMT) markers. (**A**) Wound-healing migration assay in MKN74 WT and in biglycan KO cells. The loss of biglycan decreases cell migration capacity with a concomitant increase of E-cadherin (bottom images, magnification at 200×). (**B**) Treatment with 5 μg/mL of commercial purified biglycan is able to increase cell migration in the NCI-N87 biglycan-negative cell line. Exogenous biglycan is also able to promote a mesenchymal-like phenotype with more elongated spindle-like cells. Images magnification at 200× (**C**) Migration capacity of biglycan KO cells upon exogenous treatment with commercial purified biglycan or secretome from MKN74 WT (biglycan positive). Both treatments are able to restore migration capacity of KO cells and to promote a mesenchymal-like phenotype of cells. Images magnification at 200× (**D**) Western blot analysis of epithelial (E-cadherin) and mesenchymal (N-cadherin and Nanog) markers with or without biglycan treatment. (**E**) Expression levels of epithelial markers (*CDH1* or *E-cadherin*) as well as mesenchymal markers (*CDH2* or *N-cadherin*, *SNAI1, ZEB1, ZEB2, NANOG, POU5F1,* and *DPP4*) were examined by qRT-PCR. Biglycan KO cells present increased expression of *CDH1* and decreased expression in mesenchymal markers such as *CDH2*, *SNAI1,* and *ZEB2*. Three independent analysis were performed in duplicate. (**F**) In silico analysis of epithelial and mesenchymal genes in human stomach cancer samples (STAD-TCGA, *n* = 408) using the Gene Expression Profiling Interactive Analysis (GEPIA) program corroborating the in vitro findings. In vitro data are presented as the mean ± standard error of the mean (S.E.M.) of at least three independent experiments. *, *p* value < 0.05; **, *p* value < 0.01; ***, *p* value < 0.001.

Based on the *in silico* functional annotations, we further explored the in vitro role of biglycan expression in GC cell migration and EMT process. Using our MKN74 cell line model, we observed that the loss of biglycan markedly decreased the migration capacity of cells when compared to WT cells (Figure 4A). Accordingly, the GC cell lines negative for biglycan exhibit a higher migration capacity when exposed to exogenous commercial biglycan (5 µg/mL) (NCI-N87 ctr vs. BGN: *p* value < 0.001; MKN45 ctr vs. BGN: *p* value < 0.001) (Figure 4B and Figure S5A). Further, and in order to evaluate whether the cell migratory capacity of biglycan KO cells can be restored, we treated both clones with exogenous commercial biglycan (5 μg/mL) and secretome from WT cells (containing the secreted form of biglycan) (Figure 4C). An increase in the migration capacity of both KO clones was observed either in the treatment with exogenous commercial biglycan (KO.5: *p* value < 0.05; KO.12: *p* value < 0.01) or with secretome from WT cells (KO.5: *p* value < 0.001; KO.12: *p* value < 0.001). The same effect was observed in the biglycan negative cell line NCI-N87 that was treated with MKN74 WT secretome, while no differences were observed in cell migration when NCI-N87 cells were treated with secretome from MKN74 KO clones (Figure S5B). These data indicate that secreted biglycan plays a key role in GC cell migration.

Interestingly, we observed that biglycan KO cells, when treated with exogenous biglycan or with secretome from WT cells, showed remarkable alterations in cell morphology (Figure 4C). The same is true when biglycan negative GC cells are exposed to exogenous commercial biglycan (Figure 4B). In general, the cells exhibited a more elongated cell shape (spindle like) with the formation of protrusions resemble a mesenchymal phenotype (Figure 4B,C). Thus, we want to evaluate the expression of epithelial to mesenchymal transition (EMT) markers. At protein levels, we found a slight increase of the epithelial marker E-cadherin (Figure 4A,C) and a decrease in the mesenchymal marker Nanog in KO cells (Figure 4D and Figure S4). At mRNA levels, the KO cells were characterized as having an increased expression of the epithelial marker E-cadherin (*CDH1*) and a concomitant decrease of several mesenchymal markers, including N-cadherin (*CHD2*), *ZEB1*, *ZEB2*, *SNAI1*, *NANOG*, *DPP4,* and Oct3/4 (*POU5F1*) (Figure 4E). This gene expression profile of KO cells is compatible with a mesenchymal–epithelial transition (MET) phenotype. The same genomic profile was observed in GC patients in the TCGA dataset (*n* = 408), in which *BGN* was inversely correlated with epithelial markers (*CDH1*: Spearman R = −0.38, *p* value < 0.001; *CKR19*: Spearman R = −0.27, *p* value < 0.001) and directly correlated with the mesenchymal gene signature (*VIM, SNAI1, SNAI2, TGFB1, MMP2, MMP14, CDH2, ZEB1, ZEB2,* and *FN1:* Spearman R > 0.5, *p* value < 0.001) (Figure 4F). Importantly, KO cells, when exposed to exogenous biglycan, showed a recovery of the mesenchymal marker Nanog (Figure 4D and Figure S4). However, no differences were observed in the E-cadherin and N-cadherin expression. The time point used (4 h) may not be enough to assess the turnover of E and N-cadherin expression and longer biglycan exposure time experiments should be performed.

### 3.5. Loss of Biglycan Promotes Cell Death by Apoptosis, and BGN Expression Interferes with Anti- and Pro-Apoptotic Gene Regulation in GC Patients

**Figure 5:**
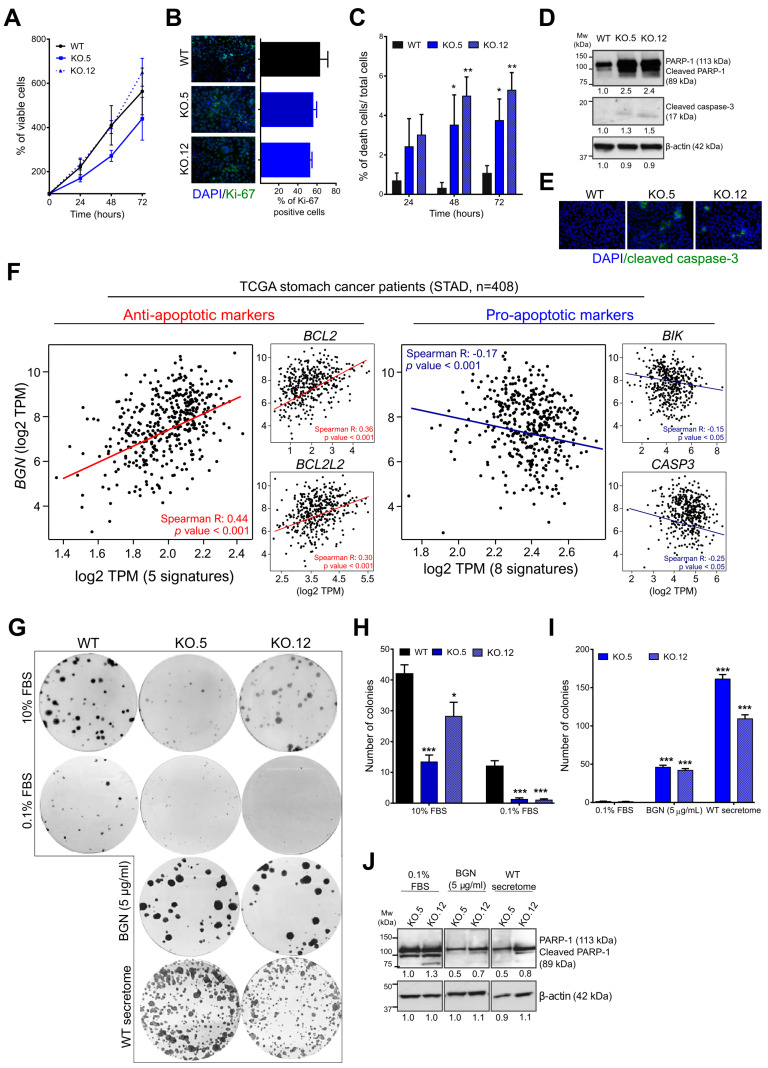
Loss of biglycan promotes GC cell death via induction of pro-apoptotic markers. (**A**) Trypan blue exclusion assay of viable cells over time. Loss of biglycan expression (clones KO.5 and KO.12) did not affect cell viability when compared to WT cells. (**B**) Ki-67 immunofluorescence (left panel) and percentage of ki-67 positive cells (right panel) relative of total amount of cells (DAPI stained cells). Images magnification at 200× (**C**) Percentage of cell death overtime assessed by trypan blue exclusion assay. Biglycan KO cells showed an increase of cell death comparing to WT control. (**D**) Immunoblot showing an increase of cleaved-PARP1 and caspase 3 in biglycan KO cells (clones KO.5 and KO.12). (**E**) Immunofluorescence analysis of cleaved-caspase 3 (green) and counterstained with DAPI to visualize the nuclei (blue). Cleaved-caspase 3 was only observed in KO cells. Images magnification at 200× (**F**) In silico analysis (TCGA, *n* = 408) demonstrating that GC patients with high levels of *BGN* expression also present a gene signature that is associated with increased anti-apoptotic markers (*BCL2, BCL2L2, BCL2A1, IQSEC2, BCL2L1)* and inversely correlation with pro-apoptotic gene signatures (CASP3, *CASP6, CASP5, CASP8, CASP10, FASN, BAK, BIK*). Gene correlation was performed using the Gene Expression Profiling Interactive Analysis (GEPIA) program. (**G**) Colony formation assay was used to study the effect of biglycan in the capacity of cells to grow at low densities and under starvation conditions (death stimuli). Exogenous biglycan (commercial purified biglycan or secretome from MKN74 WT cells) is able to restore the capacity of KO cells to form colonies. (**H**) Colony quantification in the presence or absence of serum. (**I**) Colony quantification after treatment with purified biglycan or with secretome from MKN74 WT cells. (**J**) Western blot analysis of cell death (PARP-1 cleavage) with or without treatment with exogenous biglycan. In vitro data are presented as the mean ± standard error of the mean (S.E.M.) of at least three independent experiments. *, *p* value < 0.05; **, *p* value < 0.01; ***, and *p* value < 0.001.

Our in silico analysis also indicates a putative role of biglycan in cell proliferation and cell death (Figure 2). Hence, we assessed the in vitro role of biglycan in cell viability/proliferation by trypan blue exclusion assay and Ki-67 immunostaining. We found that a loss of biglycan did not affect GC cell viability (Figure 5A) and proliferation rates (Figure 5B). However, we found a significant increase of cell death over-time in biglycan KO clones when compared with WT cells (Figure 5C). Our previous results indicate that biglycan is associated with the regulation of apoptotic processes (Figure 2B), as such we investigate the expression of apoptosis-related proteins by western blot. We found an increase in cleaved-PARP1 and caspase 3 in the KO cells, while being absent in the WT cells (Figure 5D,E and Figure S4). The gene expression of apoptosis-related genes in GC human samples showed a positive correlation between *BGN* and anti-apoptotic gene signatures (*BCL2, BCL2L2, BCL2A1, IQSEC2,* and *BCL2L1)* and an inverse correlation with pro-apoptotic gene signatures (CASP3, *CASP6, CASP5, CASP8, CASP10, FASN, BAK,* and *BIK*) (Figure 5F). Together, these results indicate that biglycan can modulate anti- and pro-apoptotic markers to sustain cancer cell viability while avoiding cell death.

### 3.6. Exogenous Biglycan Protein Stimuli is Able to Restore GC Cell Survival Capacity

In order to understand whether biglycan is, indeed, a key player in GC cell survival, we performed colony formation assay (Figure 5G). With this assay, we are able to evaluate the capacity of cells to form colonies at low densities (cell survival capabilities). We observed that the loss of biglycan significantly impairs the capacity of cells to form colonies when compared to WT cells (WT vs. KO.5, *p* value < 0.001; WT vs. KO.12, *p* value < 0.05) (Figure 5G,H). Importantly, we observed that, under unfavorable conditions (0.1% FBS, starvation), the reduced capacity to grow and form colonies was preserved in the biglycan KO models vs. WT cells (Figure 5G,H). To further assess whether biglycan could restore cell survival capabilities under adverse conditions (0.1% FBS), the KO cells were treated with 5 µg/mL of purified biglycan protein or with secretome from WT cells (Figure 5G). We found that both treatments were able to restore the ability of KO cells to form colonies (both treatment with a *p* value < 0.001) (Figure 5I). This phenotype was in agreement with a decrease in PARP1-cleavage, or cell death, in the KO cells that were treated with purified biglycan or with secretome from WT cells (Figure 5J and Figure S4). Interestingly, we observe that cells that are treated with commercial biglycan (5 µg/mL) have a lower number of colonies when compared with cells exposed to secretome from WT cells presenting, however, a drastic increase in the size of the colonies formed was observed (Figure 5G). Together, our findings suggest that biglycan has a protective effect in GC cell survival.

### 3.7. In Vivo Biglycan Angiogenic Potential of Cancer Cells

**Figure 6:**
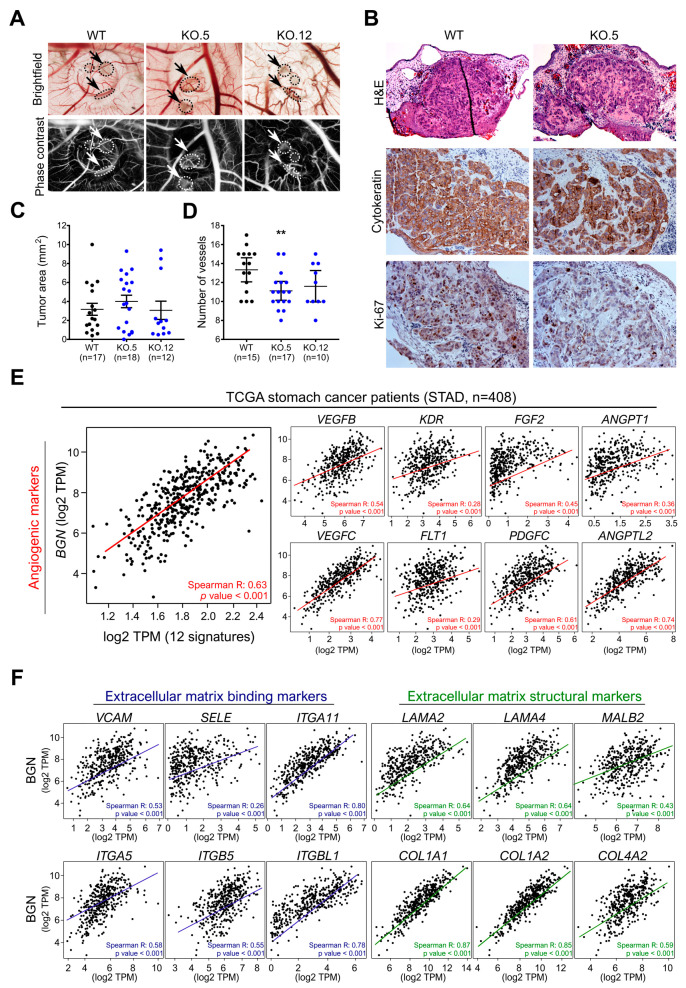
Effect of biglycan in in vivo angiogenesis. (**A**) Representative images of tumors formed in the in vivo CAM by MKN74 cell models (WT and biglycan KO clones—KO.5 and KO.12). Phase contrast was used to better visualize tumor *foci* and vessels. MKN74 form multiple tumor *foci* (arrows/circles). Magnification at 20×. (**B**) Histological images of the formed tumors, cytokeratin staining confirming the presence of human epithelial tumors in the CAM. Ki-67 expression analysis was used to assess tumor aggressiveness. Histologically, tumors formed by KO cells present a less cohesive-like tumor mass with increased extracellular matrix stiffness. Hematoxilin & Eosin (H&E) staining images at 100X magnification, cytokeration and Ki-67 images at 200× magnification. (**C**) Quantification of the tumor area (mm^2^) with no significant differences in the tumors being derived from the different cell lines. (**D**) Number of new vessels (less than 20 μm in diameter) formed towards the inoculation site. biglycan KO inoculated cells showed less capacity to form new vessels when compared to WT biglycan-positive tumors. (**E**) In silico gene analysis in GC tissues samples (TCGA, *n* = 408) showing that *BGN* was strongly positively correlated with angiogenic markers (*VEGFB, VEGFC, KDR, FLT1, FGF2, PDGFC, ANGPT1, ANGPT2, ANGPTL2, ANGPTL1, ANGPTL4,* and *ANGPTL7*)**.** (**F**) In silico analysis demonstrating that *BGN* is positively correlated with ECM binding (*VCAM1, SELE, ITGA11, ITGA5, ITGB5, ITGBL1*) and ECM structural (*LAMA2, LAMA4, LAMB2, COL1A1, COL1A2,* and *COL4A2*) genes in GC samples (TCGA, *n* = 408). Gene correlation was performed using the Gene Expression Profiling Interactive Analysis (GEPIA) program. **, *p* value < 0.01.

We assessed the capacity of GC cells in tumor formation and its angiogenic potential using the chick embryo choriallantoic membrane (CAM) xenograft model in order to investigate the role of biglycan expression in vivo. We observed that both tumors formed by WT or biglycan KO cells grow in multiple foci and not as a single tumor mass at the injection site (Figure 6A, arrows/circles). This phenotype is characteristic of the highly invasive nature of the MKN74 cell line. We characterized the formed tumors by histological analysis using H&E staining, human epithelial cytoteratine marker, and the proliferation marker Ki-67 (Figure 6B). We did not observe differences in the size of tumors that formed by WT or KO cells (Figure 6C); however, we observed a decrease in the number of Ki-67 positive cells in the biglycan KO derived-tumors (Figure 6B), pointing to a role of biglycan in cell proliferation.

Regarding the angiogenic potential of inoculated cells, we observed that KO cells promote the formation of a smaller number of new vessels (less than 20 μm in diameter) when compared to WT cells (WT vs. KO.5: *p* value < 0.01; WT vs. KO.12: *p* value = 0.057) (Figure 6D). This preliminary data suggested that biglycan might have a functional role in promoting angiogenesis in GC. Using an in silico approach, we found that, in GC tissue samples, *BGN* was strongly positively correlated with angiogenic markers (*VEGFB, VEGFC, KDR, FLT1, FGF2, PDGFC, ANGPT1, ANGPT2, ANGPTL2, ANGPTL1, ANGPTL4,* and *ANGPTL7*) (Figure 6E). The positive correlation between *BGN* and all of these markers corroborates the potential role of biglycan in promoting angiogenesis in GC. Importantly, we also observed that tumors formed by WT cells present less ECM stiffness when compared to biglycan KO tumors, characterized by a diffuse and less cohesive cell pattern of the tumors (Figure 6B). These alterations in the ECM stiffness are also in agreement with the above-mentioned potential *BGN* functional role in matrix organization/adhesion (Figure 2), and validated in GC samples by the positive correlation between *BGN* and genes that are involved in ECM binding and structure, including integrins, selectins (*VCAM1, SELE, ITGA11, ITGA5, ITGB5,* and *ITGBL1*), laminin, and collagen (*LAMA2, LAMA4, LAMB2, COL1A1, COL1A2,* and *COL4A2*) (Figure 6F).

## 4. Discussion

The present study describes, for the first time, that up-regulation of biglycan is a common event in different GC cohorts and a novel biomarker of poor prognosis in patients diagnosed with advances stages of the disease (stage III and stage IV). We also provide additional evidence that biglycan modulate oncogenic gene signatures in GC samples being associated with increased GC cell motility, survival, and angiogenic capacity both in vitro and in vivo.

The small leucine-rich proteoglycan biglycan has been described to be up-regulated in several cancer types and to be associated with tumor progression and worse prognosis of patients [22,25,47,48,49]. We observed, in six independent GC cohorts (*n* = 684 GC and *n*= 211 normal samples), that the mRNA *biglycan* (*BGN*) levels are highly expressed in GC tissues when compared with normal tissues. In a pair-wise analysis, we found a constant up-regulation of *BGN* expression in all GC samples *versus* adjacent normal tissues, indicating a potential role in GC progression. Indeed, high levels of *BGN* were associated with tumor aggressiveness, such as tumor advanced stages, lymph node invasion, and the presence of metastasis. Moreover, patients with high levels of *BGN* expression had a significant and independent shorter survival than patients whose tumors expressed low *BGN* levels; a result that was obtained in all data sets (total of 1844 GC patients with survival data). These findings are in agreement with immunohistochemical studies showing that high levels of cytoplasmatic biglycan staining are associated with GC tumor invasion and worse overall survival [28,29]. Importantly, we have described that the prognostic value of *BGN* is specific for GC patients that were diagnosed in stage III and IV, but not with early stages of disease (stage II). Together, our results uncovered biglycan as a potential new prognostic GC biomarker, which can be easily translated into a clinical setting and open the opportunity for the development of a new targeted therapy for GC patients.

Recognizing that data from in silico databases are heterogeneous (containing information from both tumor and stromal cells), which can influence the levels of *BGN* expression, we have performed an immunohistochemistry analysis in a small cohort of GC tissues. We observed that the biglycan protein is mostly absent in normal gastric glands and highly expressed in metaplasia and in tumor samples. Importantly, we were also able to demonstrate that biglycan is present in the cytoplasm of GC cells and the stroma. Although we were not able to discriminate the origin of the extracellular biglycan (cancer or stromal cells), we hypothesize that biglycan expression in tumor samples is a direct consequence of the tumorigenic progression, since it was not observed in normal counterparts. Moreover, the presence of biglycan in the extracellular matrix opens a new window of opportunities to study its clinical value as a non-invasive biomarker for GC diagnosis and prognosis [50]. Until now, all of the clinical studies involving the immunohistochemical detection of biglycan protein in GC tissues only show biglycan staining in the cytoplasm [28,29]. It is known that the mature/fully glycosylated biglycan protein, with the two chondroitine GAG chains attached to its N-terminus, is secreted by cells and integrated into the ECM [17,18]; this way biglycan staining is also expected in the ECM. The immunodetection of biglycan only at the cytoplasm indicates that the antibody implemented in these studies is only able to detect the immature form of biglycan (non-glycosylated). The main reason for this problem is the difficulty in studying the huge complexity of proteoglycans. The extreme complexity of GAGs attached to the core protein can block antibody recognition, leading to a misinterpretation of the results. Indeed, we have demonstrated that the secreted mature biglycan protein by GC cell lines is only detected after GAG removal by enzymatic digestion with chondroitinase ABC, which is in accordance with a previous study in endothelial cells [31]. As such, there is a lack of information concerning full functional biglycan at the protein level in GC tissues, and this warrants further investigation.

The exact role of biglycan in GC progression is still unknown. Thus, we investigated gene signatures that are associated with the presence or absence of *BGN* expression in 408 stomach cancers from the TCGA dataset. We found that the gene signature associated with *BGN* expression was enriched in cancer-associated pathways, such as regulation of cytoskeleton, cell migration, apoptosis, ECM adhesion, and structural organization and angiogenesis. Using the first established biglycan KO cell model, using the CRISPR/Cas9 system, we demonstrated that GC cells with a loss of biglycan present drastic phenotypic alterations that are characterized by increased cell size, with a concomitant increased expression of the epithelial markers and the downregulation of several mesenchymal markers, such as Nanog. In GC clinical samples, we observed that patients with high levels of *BGN* expression were also associated with a mesenchymal signature. These results were compatible with lower cell migration capacity being observed in biglycan KO cells when compared with WT control cells (biglycan positive). Importantly, we demonstrated that exogenous stimuli with purified biglycan protein or with secretome from biglycan-positive cells are able to restore the expression of the Nanog mesenchymal marker as well as the cell migration capabilities on biglycan-negative cell lines (NCI-N87, MKN45, and MKN74 biglycan KOs). These results indicate a key role of biglycan in GC cell motility and possibility in EMT signaling. Our results are consistent with the work of Hu et al. [29], which demonstrated an increased cell invasion in the SGC-7901 GC cell line with biglycan overexpression. Previous studies in colon, bladder, and endometrial cancers also provided evidences that biglycan promotes cancer cell migration, invasion [21,23,51], and proliferation, which ultimately result in increased resistance to 5-fluorouracil chemotherapy [52].

Based on our in silico and in vitro analysis, in which biglycan was shown to be associated with cell death regulation, we hypothesized that biglycan has a role in GC cell survival. Although biglycan has been described to modulate cancer cell proliferation [23,51], no differences were observed in the in vitro proliferation rates of our GC cell models. However, we observe a significant decrease in the Ki-67 staining in vivo tumors formed by biglycan-negative cells. Furthermore, in the in vivo context, tumors formed by biglycan-negative cells lead to a decrease of the ECM cohesive phenotype that can be associated with a decrease cancer cell survival [53]. In fact, Andrlová et al. [25] described that biglycan is able to modulate ECM organization/structure by up-regulating the expression of the integrin-β1 adhesion molecule, which, in turn, increases the matrix stiffness and modulates the tumor-related survival. Here, we also demonstrated that *BGN* is strongly associated, in GC samples, with several ECM gene signatures, including laminin, collagen, and integrin gene expression. The differences between the in vitro and in vivo proliferation analysis lead us to suggest that the role of biglycan, as an ECM proteoglycan, in cancer survival/proliferation, may be context-dependent. Moreover, the use of additional in vivo models to study tumor growth, as mice models, should also carried out to determine the effect of biglycan expression in a longer experiment.

We demonstrated that GC cells without biglycan expression (KO model) had a decreased capacity to survive and form colonies at low densities (100 cels/mL). In agreement, we found that a loss of biglycan promotes GC cell death, apoptosis, with an increased expression of cleaved-PARP1 and caspase 3. Similar findings were found in GC samples, where patients with high levels of *BGN* expression were positively correlated with a protective gene signature of anti-apoptotic markers and inversely correlated with pro-apoptotic gene signatures. These results indicate that biglycan plays a major role in the survival of cancer cells. Indeed, biglycan KO cells under cell death stimuli conditions, such as starvation (0.1% FBS), stop colony formation, which is restored after supplementation with commercial purified biglycan or with WT secretome (biglycan-positive). This phenotype is in agreement with the reduced cleaved-PARP1 expression in KO cells that were exposed to exogenous biglycan. Interestingly, the number of colonies formed with biglycan stimuli from a commercial purified form showed to have a bigger area when compared with colonies that formed with WT secretome. We believe that the differences between these two treatments were due to the presence of growth factors in the secretome, leading to an increase of cell survival and, consequently, an increased number of colonies.

One of the major functions that is associated with biglycan in cancer is its potential to modulate cancer cell angiogenesis and metastasis formation [24,26,27,30]. The overexpression of biglycan is able to increase the number of metastasis in endometrial cancer and melanoma mice models [23,26], mainly due to an increase in the angiogenic capacity of tumor endothelial cells [23,27]. Likewise, biglycan was also associated with in vitro tubular angiogenesis by activating the TLR signaling pathway and inducing VEGF expression in colon and gastric cancer [24,30]. Using the in vivo angiogenesis CAM model, we found that biglycan KO cancer cells lead to a decrease in the number of blood vessels when compared with WT tumors. We hypothesize that biglycan is able to modulate angiogenic signaling cascades that will, in turn, increase the expression of several angiogenic gene signatures, as observed in clinical human GC samples. The potential role of biglycan in cell survival, migration, and tumor angiogenesis is a starting point for a major role in GC metastasis. The new vessels formed provide oxygen and nutrients to tumor cells and the viability capacity will enable their colonization at the metastatic niche.

## 5. Conclusions

The data that are presented here allowed for the identification of biglycan as a potential prognostic and therapeutic alternative for advanced GC patients. Importantly, the present study demonstrated that secreted biglycan is associated with increased cell motility, angiogenesis, and survival capabilities in GC. These novel findings provided evidence regarding the biological role of biglycan as a key modulator of GC aggressiveness and probably in the metastatic cascade. Our results set the ground for future research aiming to target biglycan in therapeutic approaches directed at tackling cancer progression.

## Figures and Tables

**Table 1:** Multivariate analysis of *BGN* expression and overall survival in stomach cancer cohort from The Cancer Genome Atlas (STAD-TCGA) gastric cancer cohort (*n* = 323).

Clinical Parameter	HR	95% CI	*p* Value (Cox)
*BGN*	2.219	2.056–2.408	0.007
Stage II	1.715	0.803–3.665	0.164
Stage III	2.695	1.331–5.458	0.006
Stage IV	4.613	1.802–11.809	0.001
Age	1.026	1.007–1.045	0.008
Gender (male)	1.085	0.743–1.585	0.673

## Data Availability

Public available datasets used in this study can be found at: https://www.cbioportal.org/, http://kmplot.com/analysis/index.php?p=service&cancer=gastric, and at http://gepia.cancer-pku.cn/.

## Supplementary Materials

The following are available online at https://www.mdpi.com/2072-6694/13/6/1330/s1, Figure S1: Biglycan knock out (KO) gRNA validation by CRISPR/cas9 technology, Figure S2: High levels of mRNA *BGN* (*biglycan*) are associated with tumor aggressiveness and poor survival in GC patients, Figure S3: Immunodetection of biglycan in GC total cell lysate and genomic validation of biglycan knock out (KO) cellular models, Figure S4: Uncropped western blots figures. Figure S5: Biglycan-negative GC cells exposed to exogenous biglycan protein present increased cell migration capacity.
