# Supplementary material for: The Extracellular Small Leucine-Rich Proteoglycan Biglycan Is a Key Player in Gastric Cancer Aggressiveness
Source: Cancers (Basel). 2021 Mar 16;13(6):1330. doi: 10.3390/cancers13061330 (PMC8001774; doi:10.3390/cancers13061330)
Supplement: Supplementary file 1 [file cancers-13-01330-s001.pdf]

cancers

MDPI

Article

The Extracellular Small Leucine-Rich Proteoglycan Biglycan is a Key Player in Gastric Cancer Aggressiveness

Filipe Pinto, Liliana Santos-Ferreira, Marta T. Pinto, Catarina Gomes and Celso A. Reis

Supplementary Figures

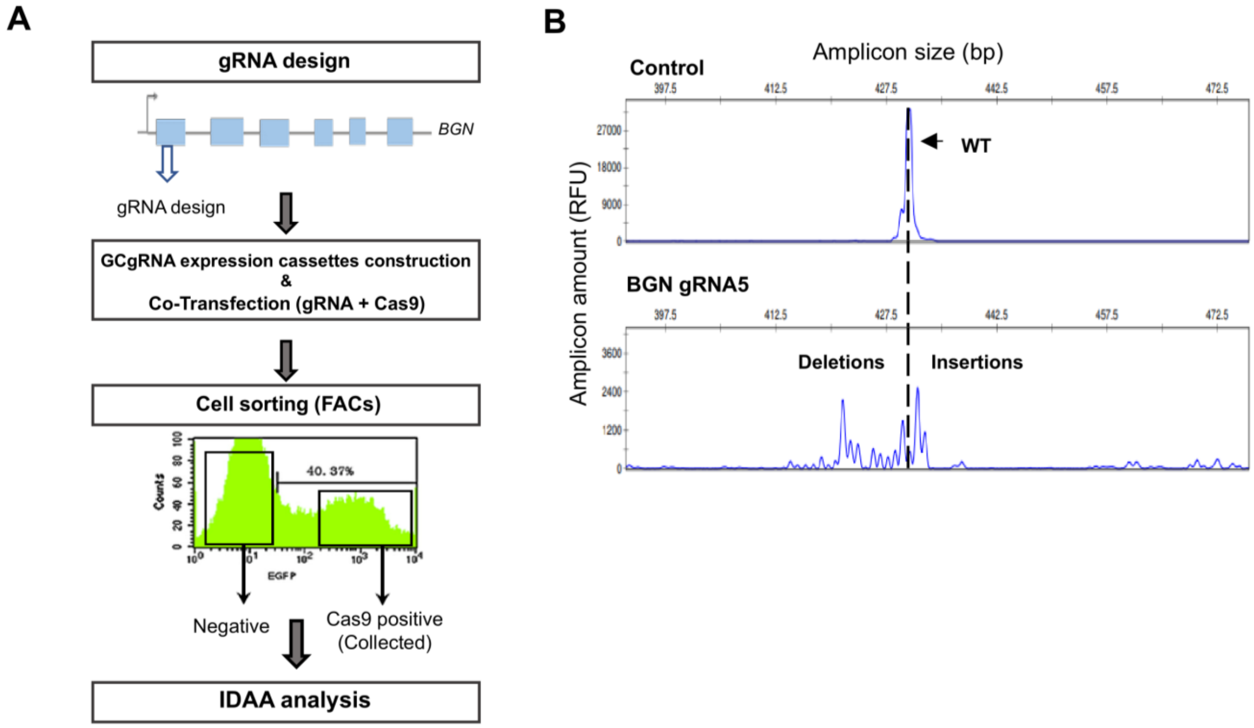

Figure S1. Biglycan knock out (KO) gRNA validation by CRISPR/cas9 technology accordingly to [39,40]. **(A)** Scheme of the CRISPR/cas9 system used to validate the biglycan KO clones: i) six gRNAs targeting exon 1 and 2 of *BGN* gene were design using DESKGEN (https://www.deskgen.com/landing/); ii) the GCgRNA expression cassettes were constructed using a tri-primer amplification protocol and co-transfected with a plasmid containing CAS9 in the HEK-293 cell lines; iii) Cas9 positive cells were sorted and indels detected by Indel Detection by Amplicon Analysis (IDAA). **(B)** IDAA profile of control DNA (WT cells) and DNA from cells transfected with selected gRNA5. The gRNA 5 was able to promote both base pairs insertions and deletions (indels) in *BGN* target site.

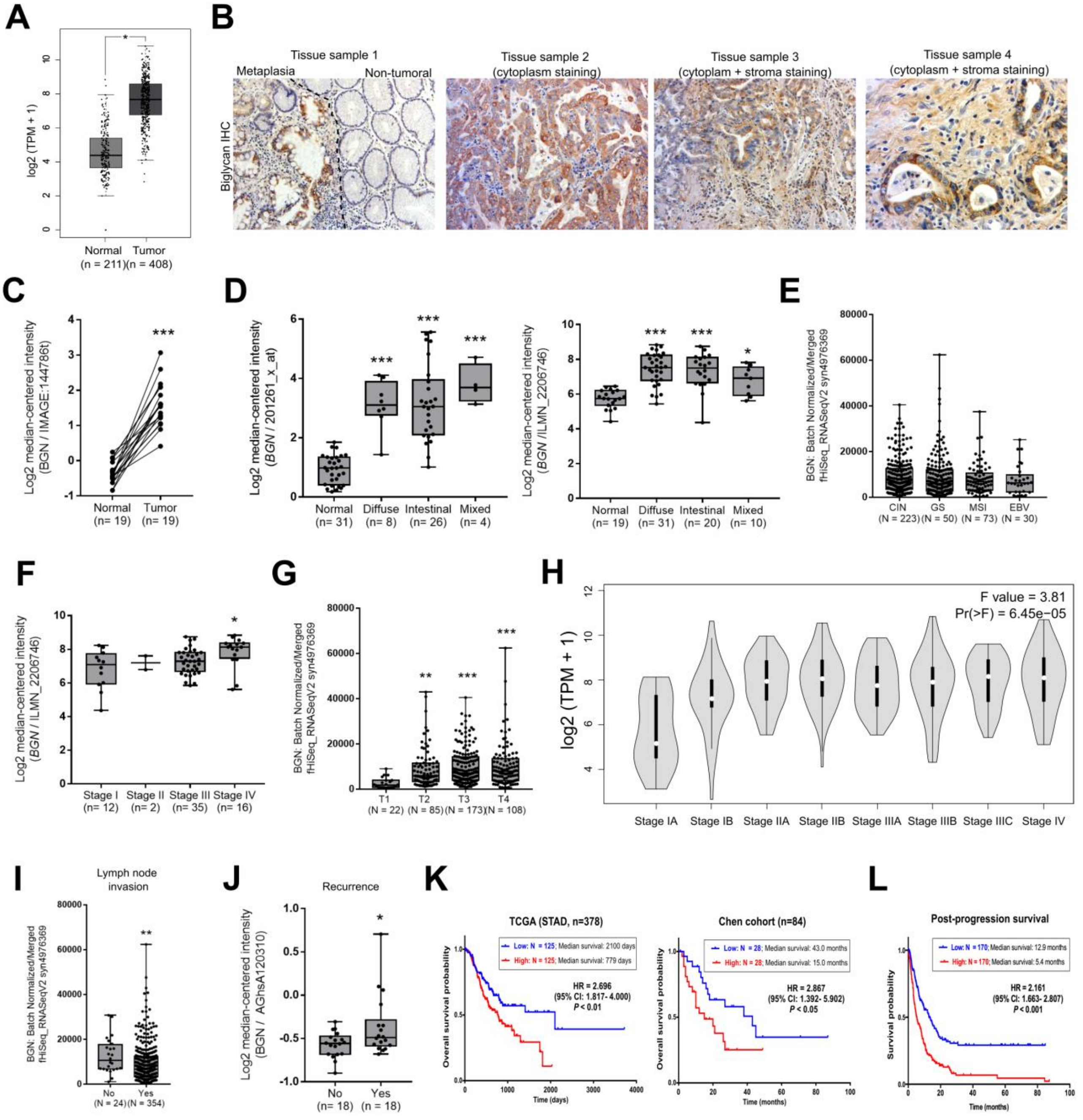

Figure S2. High levels of mRNA *BGN* (*biglycan*) are associated with tumor aggressiveness and poor survial in GC patients. **(A)** Expression levels of mRNA *BGN* in the STAD-TCGA cohort (tumor, n = 408 vs normal, n = 211). **(B)** Representative images of biglycan immunohistochemistry in GC tissues (4 cases). Biglycan is mostly absent in normal gastric glands (tissue sample 1, 200×) and it is present at both cancer cell cytoplasm and in the tumor stroma (tissue sample 2, 200×; tissue sample 3, 200×; and tissue sample 4, 400×). **(C)** *BGN* expression in patient tumor and normal paired-wise samples (Chen cohort). **(D)** *BGN* expression by Lauren subtype classification (left: DErrico cohort; righ: Cho cohort). **(E)** *BGN* expression by TCGA molecular subtype (STAD-TCGA cohort). CIN, chromosomal instability; GS, genomically stable; MSI, microsatellite instability; and EBV, Epstein-Barr virus subtype. **(F, G)** *BGN* expression by tumor stage in the Cho and TCGA cohort, respectively. **(H)** *BGN* expression subdivided by detailed tumor stage (STAD-TCGA cohort). **(I)** *BGN* mRNA levels are elevated in patients with presence of lymph node invasion (STAD-TCGA cohort) **(J)** and recurrence (Takeno cohort). **(K)** Kaplan-Meier analysis between *BGN* mRNA levels (STAD-TCGA and Chen cohort) and GC overall survival. The categorization of patients' samples was assigned into low (first tercile, lowest 33.3%) and high (third tercile, highest 33.3%) subgroups according to the levels of *BGN* mRNA expression. **(L)** Post-progression

survival analysis in the GEO cohort. Hazard ratios (HR) with 95% confidence intervals (CI) are shown. *, p value < 0.05; **, p value < 0.01; ***, p value < 0.001.

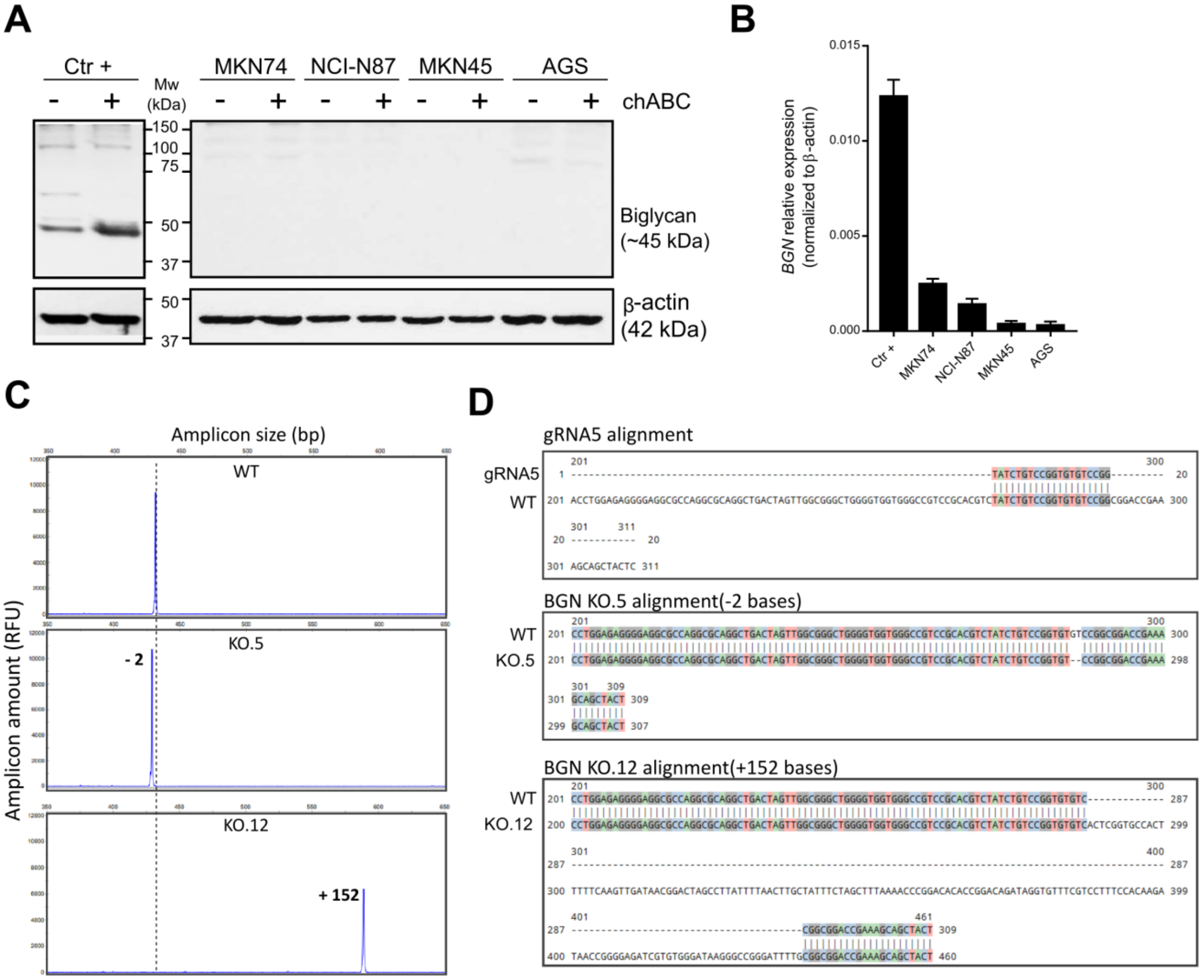

Figure S3. Immunodetection of biglycan in GC total cell lysate and genomic validation of biglycan knock out (KO) cells. **(A)** Biglycan characterization in total cell lysates samples from an intestinal cancer cell line Caco-2 (positive control) and from four GC cell lines (MKN74, MKN45, NCI.N87 and AGS) with and without chondroitinase ABC (chABC) treatment. **(B)** *BGN* mRNA levels in GC cell lines assessed by real time-PCR analysis. Data is presented as relative expression normalized for the *β-actin* housekeeping gene of three independent extractions in duplicate for each cell line. Values are means ± standard error of the mean (S.E.M.). **(C)** Genomic indels were detected in two *BGN* KO clones by RFLP analysis (KO.5: deletion of 2 (-2) nucleotide base pairs; and KO.12: insertion of 152 (+152) nucleotide base pairs). **(D)** Validation of the detected indels by DNA Sanger sequence method. Sanger sequences were alignment and analyzed using TIDE and CRISP-ID algorithm.

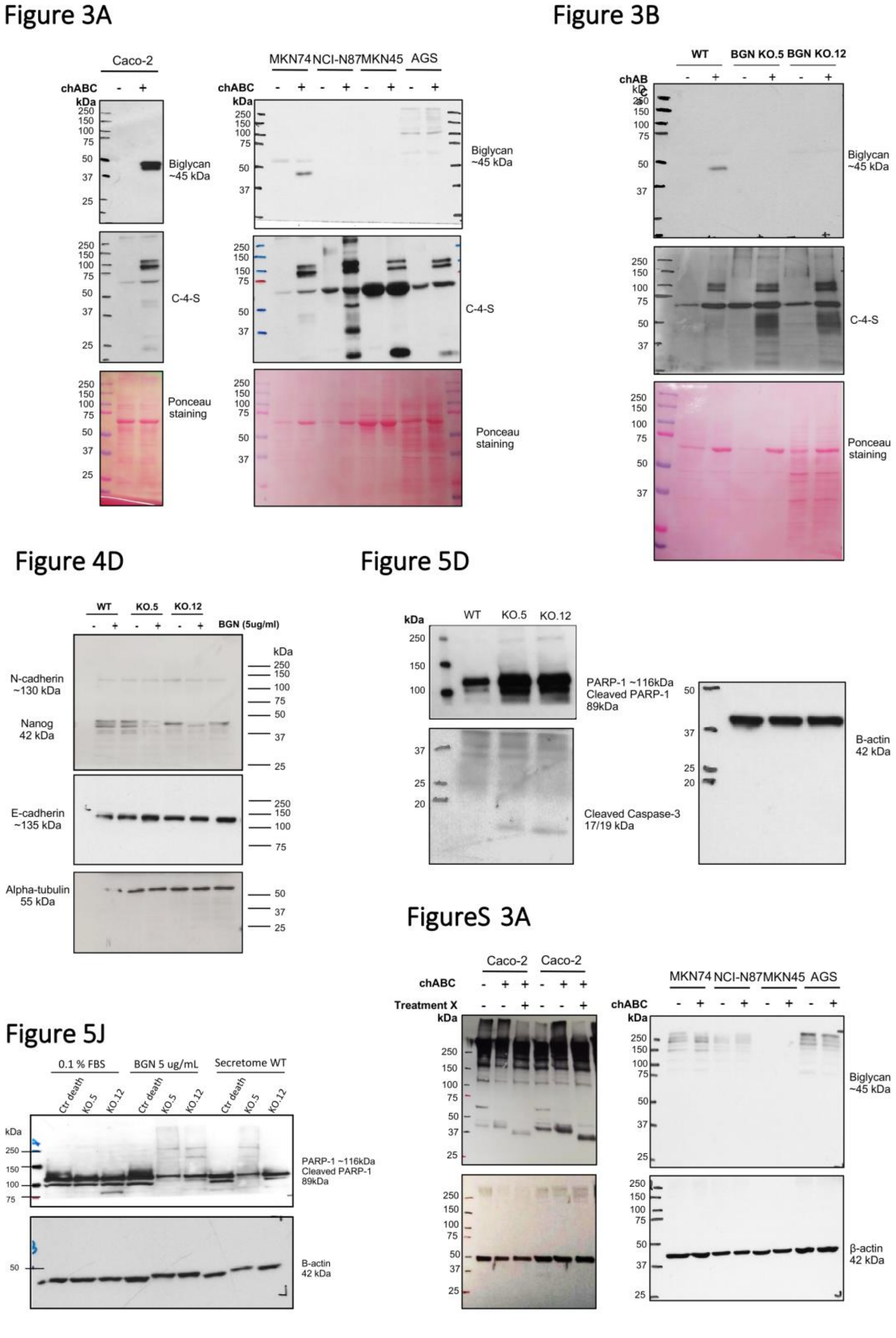

Figure S4. Uncropped western blots figures

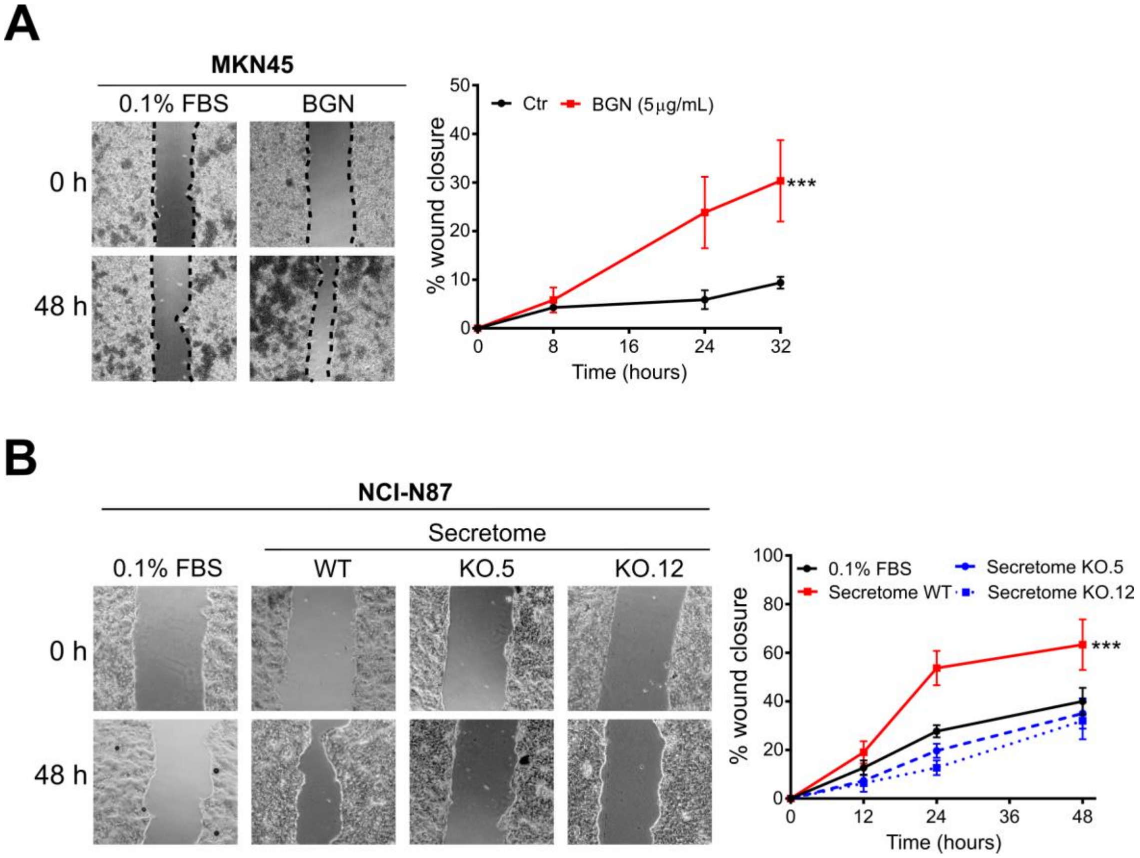

Figure S5. Biglycan-negative GC cells exposed to exogenous biglycan protein present increased cell migration capacity. **(A)** Treatment with 5 μg/ml of commercial purified biglycan is able to increase cell migration in the MKN45 biglycan-negative cell line. **(B)** Cell migration effect of biglycan-negative NCI-N87 exposed to secretomes from MKN74 WT or biglycan KO (KO.5 and KO.12). Only NCI-N87 exposed to secretome from MKN74 WT (biglycan positive) present increased capacity to migrate compared to NCI-N87 WTcontrol cells (0.1% FBS). Data are presented as the mean ± standard error of the mean (S.E.M.) of at least three independent experiments. ***, p value < 0.001.
